# Case Report: Acute hemorrhagic edema of infancy (Seidlmayer purpura) – a dramatic presentation for a benign disease

**DOI:** 10.12688/f1000research.20645.1

**Published:** 2019-10-17

**Authors:** Elena Carboni, Maria Scavone, Ettore Stefanelli, Valentina Talarico, Stefania Zampogna, Maria Concetta Galati, Giuseppe Raiola

**Affiliations:** 1 Department of Pediatrics, Magna Graecia University of Catanzaro, Catanzaro, Italy; 2 Department of Pediatrics, Pugliese Ciaccio Hospital, Catanzaro, Italy; 3 Department of Pediatric Oncology and Hematology, Pugliese Ciaccio Hospital, Catanzaro, Italy

**Keywords:** edema, leukocytoclastic vasculitis, Seidlmayer purpura, erythematous rash, children

## Abstract

We present a case of an 11-month-old girl who was referred to our unit for an erythematous rash that appeared on the face and extremities. Personal and family history was not relevant. Laboratory tests were normal. During recovery, diameter and colour intensity of the cutaneous lesions increased, but after some weeks, lesions had a self-limited resolution without any treatment. Based on clinical and laboratory findings, a diagnosis of acute hemorrhagic edema of infancy (AHEI) was made.  AHEI is a rare cutaneous leukocytoclastic vasculitis that usually affects children aged between 4 and 24 months. Etiology is unknown but almost of 75% of cases are preceded by infectious episodes, vaccinations or use of medications. In contrast to the dramatic cutaneous eruption, clinical conditions are usually optimal. Classically, AHEI is characterized by a triad of symptoms: fever, edema and purpura. Skin lesions are erythematous, annular, medallion-like, purpuric plaques that have a rapid onset and appear on the face and extremities, sparing trunk and mucosal membranes. Initially interpreted as a variant of Henoch-Schönlein purpura, now it is considered a distinct disease. In the majority of cases the disease is benign and self-limited without a visceral involvement, so a conservative approach is most often chosen.

## Introduction

Acute hemorrhagic edema of infancy (AHEI), also known as Seidlmayer purpura, is a rare cutaneous leukocytoclastic vasculitis. It was described for the first time in 1913 and currently there are more than 300 cases in the literature ^1^. Initially it has been interpreted as a Henoch-Schönlein purpura variant, but now it is considered a distinct disease. Although it has a dramatic clinical presentation, it is a benign and self-limited disease ^2^. We report the case of an 11-month-old girl who was referred to our unit for an erythematous rash appeared on the face and extremities, which was indicative of a rare but non negligible diagnosis.

## Case presentation

**Figure 1.**
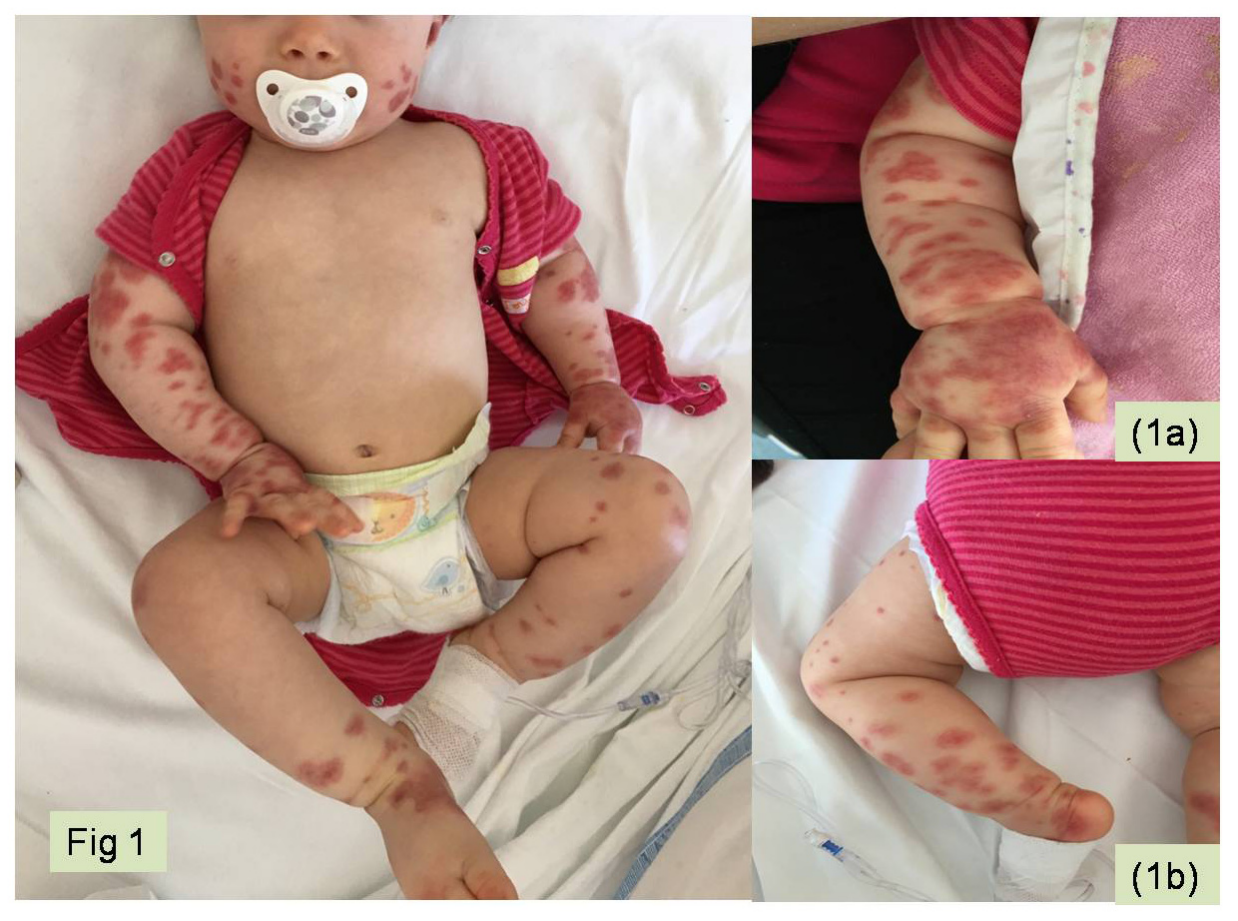
Cutaneous presentation of acute hemorrhagic edema of infancy in our case. ( **a**) Ecchymotic target-like skin lesions with edema of the hand. ( **b**) Purpuric confluent elements with a cockade pattern on the lower limbs.

An 11-month-old girl from France presented with fever and oval purpuric lesions on the face and extremities, which had appeared one hour before. In the previous week, she had bilateral conjunctivitis and gastroenteritis, treated with oral rehydration. At admission she was in good clinical condition and her vital signs were normal. Physical examination showed purpuric confluent elements with a cockade pattern on cheeks, left auricle, upper and lower limbs, particularly on distal ends, sparing trunk and back ( Figure 1). Hands appeared edematous without joint swelling or tenderness. Bilateral conjunctivitis was still present. No other physical abnormalities were observed. Complete blood count, extended biochemistry, coagulation tests (prothrombin time, partial thromboplastin time, fibrinogen and D-dimer) and urinalysis were normal with C-reactive protein of 18.9 mg/l (normal value <5 mg/l) and procalcitonin of 0.88 ng/ml (normal value <0.05 ng/ml). During the hospitalization the child maintained good clinical condition, with stable vital parameters. Dermatological lesions showed a worsening clinical outcome, with increased diameter and colour intensity during the following three days from admission. Based on clinical and laboratory findings, a diagnosis of AHEI was made. We decided to not perform any therapy and after about two weeks lesions had a self-limited resolution. The child was monitored clinically for about six months and she did not present any relapse of the disease during the follow-up period.

## Discussion

The first description of AHEI was made by Snow in 1913 ^1^. There have been approximately 300 cases reported in the literature ^2^ under a variety of denominations: Seidlmayer purpura, Finkelstein disease, rosette form purpura, medallion-like purpura, infantile post-infectious iris-like purpura and edema ^3^. AHEI is a rare cutaneous leukocytoclastic vasculitis that usually affects children aged between 4 and 24 months and it is more common in males ^2, 4^. Most cases of AHEI occur during winter, and almost of 75% of cases are preceded by infectious episodes, such as viral and bacterial infections of the upper respiratory tract, otitis media, conjunctivitis, bronchopneumonia, urinary tract infections and enteritis. Many organisms including adenovirus, varicella zoster virus, cytomegalovirus, rotavirus, herpes simplex virus, tuberculosis, streptococci, and staphylococci are associated with AHEI. Vaccinations or medications could also trigger AHEI ^5^. In this case, the patient had a week-long history of bilateral conjunctivitis and gastroenteritis, compatible with a recent viral infection. A peculiar feature of AHEI is the unusual dramatic cutaneous eruption that contrasts with good general clinical condition (normal vital parameters with normal blood tests) that allowed to exclude more serious diseases. Diagnosis is clinical and it is classically detectable by observance of the clinical triad of symptoms: fever, edema and purpura ^6^. Skin lesions are erythematous, annular, medallion-like, rosette-shaped purpuric plaques that cluster and often coalesce ^3^. These lesions have a rapid onset and appear on face and extremities, sparing trunk and mucosal membranes ^3, 4^. The edema typically occurs on feet, hands, face and auricles, and can involve the scrotum in males ^2, 7^. In the majority of cases there is no visceral involvement and the disease is benign and self-limited. However, in the literature there are descriptions of cases of renal involvement, arthralgias and two cases of intestinal involvement followed by intussusception ^2^.

Krause *et al*. suggested the following criteria for diagnosing AHEI ^8^:

- Age <2 years;
- Purpuric or ecchymotic target-like skin lesions with edema on the head and face, with or without mucosal involvement;
- Lack of systemic disease or visceral involvement;
- Spontaneous recovery within few days or weeks.

Our patient presented all of the above conditions. Laboratory data are usually normal, but leukocytosis, thrombocytosis, eosinophilia and high levels of C-reactive protein and erythrocyte sedimentation rate can be observed ^7^. Skin biopsy shows a leukocytoclastic vasculitis of the small dermal vessels characterized by infiltration of perivascular neutrophils, showing fragmentation of nuclei, resulting in fibrinoid necrosis ^9^. Differential diagnoses include urticaria, erythema multiforme, idiopathic thrombocytopenia, meningococcemia, Kawasaki disease, Sweet syndrome, Gianotti-Crosti disease, drug-induced vasculitis, child abuse and trauma-induced purpura, but Henoch-Schönlein purpura is probably the most important ^9, 10^. Initially interpreted as a variant of Henoch-Schnölein purpura, now it is considered a distinct disease with different epidemiological, clinical and pathological features ^4^.

Treatment of AHEI remains controversial; conservative management is the most frequently approach, because this disease is a self-limited condition ^4, 7, 9^. AHEI certainly represents a challenge for the pediatrician at the emergency department and it requires, at least initially, a high level of suspicion for potentially serious pathologies that need adequate, urgent treatment such as for infectious (meningococcemia) or hematological diseases (autoimmune thrombocytopenia, coagulopathies). In fact, in our case some easily laboratory tests, such as complete blood count, prothrombin time, partial thromboplastin time, fibrinogen, D-dimer, C-reactive protein and procalcitonin tests were supportive in the rapid exclusion of these conditions. This approach, together with the typical benign course of the AHEI, quickly guaranteed the exclusion of these conditions, avoiding the execution of unnecessary and/or invasive diagnostic procedures and unrequired therapies.

We reported this case because it is expression of a rare and often under-recognized disease by pediatricians. A typical feature of AHEI is the discrepancy between dramatic cutaneous involvement and good clinical conditions of the affected children. This characteristic can reassure clinicians about the absence of a serious medical condition, but it is possible only through the knowledge of the disease.

## Conclusion

We describe a case of AHEI with striking cutaneous involvement that resolved spontaneously. AHEI is an uncommon disease, often under-recognized. For this reason, it is crucial that physicians have the skill to recognize this self-limited disease to avoid parental anxiety and inappropriate procedures or therapies.

## Data availability

All data underlying the results are available as part of the article and no additional source data are required.

## Consent

We received written informed consent from the patient’s family for the publication of this manuscript.
