# Supplementary material for: Case Report: Acute hemorrhagic edema of infancy (Seidlmayer purpura) – a dramatic presentation for a benign disease
Source: F1000Res. 2019 Oct 17;8:1771. [Version 1] doi: 10.12688/f1000research.20645.1 (PMC6944248; doi:10.12688/f1000research.20645.1)
Supplement: Peer review history

# Peer review: Reviewer response for version 1

**DOI:** 10.5256/f1000research.22706.r57605

**Published:** 2019-12-27

**Authors:** Antonio Francesco Urbino

**Affiliations:** 1 Department of Pediatric Emergency, Regina Margherita Children's Hospital, A.O.U. Città della Salute e della Scienza di Torino, Turin, Italy

The case report is interesting, well written and provides complete information for paediatricians who have never met the acute hemorrhagic edema of infancy in their practice. 

 Just a few minor observations:

1. The acute hemorrhagic edema of infancy is more known as Finkelstein disease than as Seidlmayer purpura: I suggest to change the eponym both in the introduction and in the keywords.
2. The French origin of the girl does not influence the diagnosis and is redundant: please remove it.
3. In the discussion section, Finkelstein disease is said to be distinguished from Gianotti-Crosti disease, whose papular and not usually purpuric presentation is quite different: I suggest to remove it.
    
4. Authors state the the Finkelstein disease is more frequent in winter time: when did the girl arrived in the Emergency Department?

I confirm that I have read this submission and believe that I have an appropriate level of expertise to confirm that it is of an acceptable scientific standard.

# Peer review: Reviewer response for version 1

**DOI:** 10.5256/f1000research.22706.r57576

**Published:** 2019-12-09

**Authors:** Mario Bianchetti

**Affiliations:** 1 Università della Svizzera italiana, Lugano, Switzerland

The case report entitled “Acute hemorrhagic edema of infancy (Seidlmayer purpura) – a dramatic presentation for a benign disease” is interesting and rather well-presented. The following 12 changes might ameliorate the attractiveness of the report:

1. The term “Seidlmayer purpura” is popular in Germany (and perhaps in Italy). Most authors use the term “Finkelstein-Seidlmayer purpura” (Finkelstein reported his case in 1929, Seidlmayer in 1939). Please modify the manuscript accordingly.
2. Present manuscript: “…is a rare cutaneous leukocytoclastic vasculitis.”
  My suggestion: “…is a rare **small vessel** cutaneous leukocytoclastic vasculitis affecting infants 4 weeks to 24 months of age.”
3. Statement: “Initially it has been interpreted as a Henoch-Schönlein purpura variant, but now it is considered a distinct disease.” Remove this statement from the Introduction section (this issue is addressed in the Discussion section).
4. Statement: “An 11-month-old girl from France presented with fever and oval purpuric lesions on the face and extremities, which had appeared one hour before.” Please provide temperature value or at least “moderate” respectively “high” fever (furthermore: state either “axillary” or “rectal”).
5. Skin lesions are sometimes painful in Finkelstein-Seidlmayer disease. I wonder if lesions were painful in this case (important information).
6. Was pruritus present (it is usually absent in this vasculitis)?
7. Statement: “Many organisms including adenovirus, varicella zoster virus, cytomegalovirus, rotavirus, herpes simplex virus, tuberculosis, streptococci, and staphylococci are associated with AHEI.”
  My suggestion: remove “tuberculosis” (because tuberculosis has never been associated with this vasculitis after 1969).
8. Statement: “Vaccinations or medications could also trigger AHEI”. However, no cases of AHEI have been causally associated, to the best of my knowledge, with the medications.
  My suggestion: modify the statement accordingly.
9. Statement: “In the majority of cases there is no visceral involvement and the disease is benign and self-limited.”
  My suggestion: “In the vast majority of cases there is no visceral involvement and the disease is benign and self-limited.
10. The statement “Treatment of AHEI remains controversial; conservative management is the most frequently approach, because this disease is a self-limited condition” is poorly presented.
  My suggestion: AHEI is a self-limited condition. The lesions disappear within 3 weeks in ≥80% of the cases. Systemic steroids, non-steroidal anti-inflammatory drugs or antihistamines are prescribed in many cases. It is currently assumed, however, that these drugs do not alter the disease course. Once the diagnosis is established, supportive care and reassurance are advised.
11. The total length of the current report is excessive (please reduce the total length by ≥10%).
12. References:
  - Please remove references number 6 (Miorin) and number 10 (Tagliabue) because they were not published in English language (local journals).
  - Please add the following new reference: Lava *et al.* (2017 ^1^) (recent review, more up-to-date than current reference number 2.)

I confirm that I have read this submission and believe that I have an appropriate level of expertise to confirm that it is of an acceptable scientific standard.

## References

[1] : Cutaneous Manifestations of Small-Vessel Leukocytoclastic Vasculitides in Childhood. *Clinical Reviews in Allergy & Immunology*.2017;53(3) : 10.1007/s12016-017-8626-3 439-451 10.1007/s12016-017-8626-3 28836099
